# Deriving the coronary artery calcium score from computed tomography of the chest

**DOI:** 10.1590/0100-3984.2018.51.1e1

**Published:** 2018

**Authors:** Henrique Simão Trad

**Affiliations:** 1 Radiologist for Lotus Radiologia, Postgraduate Student at the Faculdade de Medicina de Ribeirão Preto da Universidade de São Paulo (FMRP-USP), Ribeirão Preto, SP, Brazil. E-mail: hsimtrad@gmail.com

There is no doubt regarding the importance and socioeconomic impact of cardiovascular diseases, especially coronary artery disease (CAD). That is not the case in scientific discussions on diagnosis, treatment, and, especially, coronary risk stratification, which is one of the pillars of cardiology. It is well-established clinical practice to use global risk scores as an initial tool in the evaluation of patients with CAD, the Framingham risk score being the most widely used. However, given the quite heterogeneous presentation of the disease, one of the main questions is whether such scores should be used alone^1^.

In the search for noninvasive complementary methods of evaluating CAD, initially with electron beam computed tomography (CT) and subsequently with new generations of multichannel CT scanners, the creation of a coronary artery calcium (CAC) score was a natural development. In addition to being noninvasive, determination of the CAC score is a robust, simple examination, its main drawbacks being the limited availability of specific equipment and the use of ionizing radiation. After years of constant accumulation of scientific data, the CAC score has proven to be solid, not only being considered useful for coronary risk stratification, with values higher than those of the clinical methods cited, but also providing important prognostic information for various clinical scenarios. According to the Second Guidelines on Cardiovascular Magnetic Resonance Imaging and Computed Tomography, issued jointly by the Brazilian Society of Cardiology and the Brazilian College of Radiology^2^, determination of the CAC score is the most accurate tool for the detection of subclinical atherosclerosis, refining the risk stratification in asymptomatic patients. In those same guidelines, the use of the CAC score in asymptomatic patients in whom the overall coronary risk is deemed intermediate was categorized as having a grade I recommendation and an A level of evidence. Therefore, in that scenario, the usefulness of the CAC score is indisputable. The Brazilian National Health Insurance Agency does not include the use of the CAC score on its minimum coverage list. This suggests that legislators are not truly acting in the best interests of the population´s health. It also leads us to believe that there is other, non-scientific, "knowledge" that pervades that decision making process.

Studies on the cardiovascular system have recently been prominent in the radiology literature of Brazil^3-6^. One study published in this issue of Radiologia Brasileira represents an innovative step forward in the understanding of the CAC score within the literature of Brazil. In that study, Pelandré et al.^7^ explored a current tendency for the CAC score to be extrapolated from chest CT scans that are not triggered by an electrocardiogram. Following a recent trend in the literature, cited in the article itself, the authors sought to determine whether data related to the relevance of the CAC score can be extracted from an examination that is much more common and comprehensive. It makes perfect sense, especially because the guidelines of the leading pulmonology, thoracic surgery, and oncology societies recommend chest CT with low-dose radiation for lung cancer screening^8^. Although some technical questions remain, the robustness of the data is undeniable, because they are based on findings in which the fundamentals of CT have been shown to be solid: spatial resolution; temporal resolution; and, in the specific case of calcifications, contrast resolution.

In a more distant future, is the CAC score as we know it today doomed to disappear? Is it within the realm of possibility that lung cancer or CAD could both be evaluated in a single screening? I do not believe that it makes sense for medicine to move in a direction in which, for technical convenience, the role of the physician is reduced or worse, abdicated. That is a perilous path that is known to have many shortcomings. The complexity of the question for the scientific method is enormous, the populations are extremely different, and the clinical settings are highly variable. Drawing the conclusion that individuals referred for coronary risk assessment might benefit, in the form of a reduction in lung cancer mortality, from a complementary screening that was not indicated for those individuals... well, you have an idea of where I am heading. It is better to imagine that, during a clinically indicated evaluation of the chest, we are also able to perform a coronary analysis-whether qualitative or (preferably) quantitative-thus precluding the need to expose the patient to additional doses of radiation in follow-up examinations that would produce similar results, as well as reducing examination times and costs. In my opinion, that would make more sense.

In view of the considerations outlined above, some things are certain. It is now unacceptable for a radiologist to allow coronary calcification identified on a CT scan of the chest to go unreported. It seems that there is a trend toward making it mandatory for the CT report to include a qualitative analysis of the coronary atherosclerosis load. We should prepare for the eventuality that, in the near future, we will be required to provide a quantitative analysis as well. However, we should not expect to be compensated for providing that analysis; for that, the road will be even more arduous that was that of producing the scientific evidence.
